# Polyphenols and IUGR pregnancies: Maternal hydroxytyrosol supplementation improves prenatal and early-postnatal growth and metabolism of the offspring

**DOI:** 10.1371/journal.pone.0177593

**Published:** 2017-05-17

**Authors:** Marta Vazquez-Gomez, Consolación Garcia-Contreras, Laura Torres-Rovira, José Luis Pesantez, Pedro Gonzalez-Añover, Ernesto Gomez-Fidalgo, Raúl Sanchez-Sanchez, Cristina Ovilo, Beatriz Isabel, Susana Astiz, Antonio Gonzalez-Bulnes

**Affiliations:** 1 Faculty of Veterinary Sciences, UCM, Madrid, Spain; 2 SGIT-INIA, Madrid, Spain; The University of Manchester, UNITED KINGDOM

## Abstract

Hydroxytyrosol is a polyphenol with antioxidant, metabolism-regulatory, anti-inflammatory and immuno-modulatory properties. The present study aimed to determine whether supplementing the maternal diet with hydroxytyrosol during pregnancy can improve pre- and early post-natal developmental patterns and metabolic traits of the offspring. Experiment was performed in Iberian sows fed a restricted diet in order to increase the risk of IUGR. Ten sows were treated daily with 1.5 mg of hydroxytyrosol per kg of feed between Day 35 of pregnancy (30% of total gestational period) until delivery whilst 10 animals were left untreated as controls. Number and weight of offspring were assessed at birth, on post-natal Day 15 and at weaning (25 days-old). At weaning, body composition and plasma indexes of glucose and lipids were measured. Treatment with hydroxytyrosol was associated with higher mean birth weight, lower incidence of piglets with low birth weight. Afterwards, during the lactation period, piglets in the treated group showed a higher body-weight than control piglets; such effects were even stronger in the most prolific litters. These results suggest that maternal supplementation with hydroxytyrosol may improve pre- and early post-natal development of offspring in pregnancies at risk of IUGR.

## Introduction

Inadequate maternal nutrition and/or placental efficiency can result in insufficient supply of oxygen and nutrients to the fetus, causing intrauterine growth restriction (IUGR) and leading to the birth of small-for-gestational-age (SGA; also known as low-birth-weight, LBW) offspring [1, 2].

In humans, LBW is associated with increased risk of perinatal morbidity and mortality, accounting for 800,000 neonatal deaths worldwide [3]. Moreover, surviving offspring are predisposed to lifelong chronic non-communicable disorders such as obesity, type II diabetes and cardiovascular diseases [4–6].

In veterinary medicine and animal production, perinatal deaths due to LBW offspring are also significant, but even more concerning are the substantial economic losses to farms. In addition to lost productivity, IUGR can reduce the value of farm products by introducing undesirable heterogeneity: low-birth-weight animals show lower growth potential, lower feed efficiency, lower meat yield and excess adiposity relative to their normal-weight littermates, giving rise to heterogeneous growth patterns, carcass conformation and meat characteristics among individuals in the same litter and feedlot [7–10]. These differences penalize the value of the product since the market requires products that are uniform in weight, conformation and composition.

In consequence, there is a strong necessity of strategies and tools for alleviating incidence and consequence of IUGR, both in human and veterinary medicine. IUGR involves not only inadequate nutrient supply but also reduced oxygenation, which causes hypoxia that increases oxidative stress and triggers low-grade inflammation [11]. The antioxidant defense system in IUGR fetuses is weakened, exacerbating oxidative stress [12–14], which in turn aggravates the effects of IUGR [15]. Previous studies in sheep have shown that the deleterious effects of oxidative status at the fetoplacental unit may be prevented by the administration of antioxidant agents (e.g.: antioxidant vitamins and melatonin), which ameliorates the antioxidant/oxidative status, improves the placental function and increases the weight and viability of the newborn [16–19].

The present trial aimed to study the effects, on pregnancies affected by IUGR, of a different source of antioxidant agents than vitamins, i.e. polyphenols. Polyphenols are common constituents of many plant-derived foods (i.e.: pieces or derivatives of fruits, vegetables and seeds), and the most abundant dietary antioxidants. Specifically, we are interested in studying the possible usefulness of the maternal supplementation with hydroxytyrosol. Hydroxytyrosol is a polyphenol present in olive fruits (and, hence, in virgin olive oil) with not only a prominent antioxidant activity, but also metabolism-regulatory, anti-inflammatory and immuno-modulatory properties [20]. These benefits are boosted by high stability, degree of absorption and bioavailability of the active substance [21]. In consequence, there is increasing clinical and epidemiological evidence on its relevance against pathologies such as cancer, cardiovascular, metabolic and neurodegenerative diseases [22].

However, possible usefulness of polyphenols for reproductive health and pregnancy is only beginning to be explored [23] Hence, we aimed to determine whether hydroxytyrosol supplementation of maternal food during pregnancy may improve pre- and early post-natal developmental patterns and metabolic traits of the offspring by using a translational swine model previously developed in our laboratory [8, 24].

## Material and methods

### Ethics statement

The study was performed according to the Spanish Policy for Animal Protection RD53/2013, which meets the European Union Directive 2010/63/UE about the protection of animals used in research. The study protocol was specifically assessed and approved by the INIA Committee of Ethics in Animal Research (report CEEA 2012/036), which is the named Institutional Animal Care and Use Committee (IACUC) for the INIA. Sows were housed in INIA animal facilities, which meet local, national and European requirements for Scientific Procedure Establishments.

### Experimental design

The study involved 20 primiparous Iberian sows that became pregnant after cycle synchronization with altrenogest (Regumate^®^, MSD, Boxmeer, The Netherlands) and insemination with cooled semen from a purebred Iberian boar.

Sows were fed a standard grain-based diet with mean content values of 89.8% of dry matter, 15.1% of crude protein, 2.8% of fat, and 12,56MJ of Metabolizable Energy ⁄Kg. From the start of the experimental period until gestational Day 35, food amount was adjusted to fulfill individual daily maintenance requirements based on data from the British Society of Animal Science [25]. On gestational Day 35, all sows were weighed and the food amount from that day until delivery was adjusted to fulfill 50% of daily maintenance requirements. This diet restriction has been previously found to affect fetal development and to induce lower birth-weight in the newborns [8, 24]. Also on gestational Day 35, sows were pair-matched according to body-weight and 10 females remained as untreated control group (group C) whilst the remaining 10 females (group HT) acted as the treated group by receiving 1.5mg of hydroxytyrosol per kg of feed each day from Day 35 of pregnancy to delivery.

### Assessment of morphological features and early post-natal development of piglets

At birth, the total number of live and stillborn piglets in each litter was recorded, together with the sex and weight of all piglets. Piglets with LBW were defined as those with a birth-weight lesser than one standard deviation of the mean value of the control littermates after adjusting for sex [26]. In addition, the percentage of piglets with body weights less than 1 kg was recorded; this weight threshold is commonly used to identify animals at higher risk of perinatal mortality [27]. All live piglets were tagged with earrings and underwent within-group fostering in order to equalize the number of piglets among sows. Piglets remained with sows in pens (one sow per pen) until weaning at 25 days-old. The number and causes of death were recorded from birth to weaning. Living piglets were weighed at 15 and 25 days-old. Average daily weight gain (ADWG) was determined for the total period of lactation as well as for two intermediate periods (from post-natal Day 0 to Day 15 and from Day 15 to Day 25). ADWG was calculated using the formula: [final weight—initial weight] / number of days.

### Assessment of body composition of piglets

At weaning, piglets were euthanized using CO_2_, stunned and exsanguinated in compliance with standard procedures stipulated in Spanish regulation RD53/2013. Back-fat depth and loin diameter were measured immediately at the P2 point, at the level of the head of the last rib, using an ultrasound machine with a multifrequency linear array probe (SonoSite S-Series, 5–8 MHz; SonoSite Inc., Bothell, WA, USA). Then, the head was separated from the trunk at the atlanto-occipital union and weighed in order to determine the ratio of head-to-body weight. Afterwards, all viscerae were removed and weighed together. Finally, major organs (brain, heart, lungs, liver, intestine, kidneys, spleen, pancreas and adrenal glands) were weighed individually for assessing possible patterns of asymmetrical IUGR. The following weight-ratios were considered: weight of brain, heart, lungs, liver, kidneys, intestine, pancreas, spleen and adrenals relative to total viscera weight.

### Assessment of the metabolic status of piglets

Blood sampling was performed, during sedation, by puncture of the *vena cava cranealis* using sterile 5-ml EDTA vacuum tubes (Vacutainer Systems Europe; Becton Dickinson, Meylan Cedex, France). Blood samples were centrifuged at 1500*g* for 15min and the plasma was stored in polypropylene vials at -20°C until assayed for determination of parameters related to metabolism of glucose and lipids.

Parameters for glucose (glucose and fructosamine) and lipids profiles (triglycerides, total cholesterol, high-density lipoproteins cholesterol [HDL-c] and low-density lipoproteins cholesterol [LDL-c]) were assessed with a clinical chemistry analyzer (Saturno 300 plus, Crony Instruments s.r.l., Rome, Italy), according to the manufacturer’s instructions.

### Statistical analyses

T-student tests were used to assess the effects of independent variables (maternal diet and piglet sex) on litter size, occurrence of IUGR and LWB and percentage of piglets below 1Kg BW). For statistical purposes, litter size was afterwards categorized in two groups (2–6 *vs* >6 piglets/litter), based on the mean litter size for the Iberian breed (6.5 piglets [28], or into three groups (2–6, 7–8 *vs* 9–10 piglets/litter) for assessing possible effects in the most prolific litters. Dependent variables related to offspring phenotype (weight, ADWG, back-fat depth, muscle diameter, organ development and metabolic indexes) were assessed using two-way ANOVA in a General Linear Model; interactions among potential confounding factors were observed and fixed when statistically significant (*P*<0.05; life time, litter size, maternal diet and offspring sex and their interaction). Differences between male and female piglets and between litter sizes in body weight, organ development and metabolic indexes were assessed using t-student test. Finally, changes over time were assessed by ANOVA for repeated measures with the Greenhouse-Geisser correction. All the results were expressed as mean ± SEM, and statistical significance was accepted from P < 0.05, while P-values between 0.05 and 0.09 were considered to indicate a tendency.

## Results

### Effects of hydroxytyrosol supplementation on birth-weight and -size and early postnatal development of the piglets

The hydroxytyrosol supplementation did not significantly affect litter size (group HT: 6.7±0.1 piglets, range of 2–10; group C: 5.8±0.1 piglets, range of 2–10), but it was related to significant increases in BW of the piglets at birth and during the postnatal period.

The group HT was characterized by a higher mean birth-weight than the group C (1.3±0.1 *vs* 1.2±0.1 Kg, respectively;, P < 0.001), lower incidence of LBW (6.3 *vs* 13.2%, respectively; P < 0.05) and lower incidence of individuals with a birth-weight lower than 1Kg (6.3 *vs* 17.0%, respectively; P < 0.05). In both groups, these percentages were numerically lower in female than in male piglets (group HT: 4.0 vs 7.9% and group C: 9.5 *vs* 18.7%, respectively; P = 0.15).

The effects of hydroxytyrosol treatment on birth-weight were determined by litter size and offspring sex (Tables 1 and 2). Overall, the group HT had higher BW values than the group C as previously described (P<0.001), the neonates were heavier in litters with a lower number than seven piglets (P<0.01) and males were heavier than females (P<0.05). As a result of both of these influences, male piglets in the group HT were significantly heavier than C male counterparts and HT female littermates (P<0.05). Females in the hydroxytyrosol group tended to be heavier than female controls (P = 0.06), but this difference did not achieve statistical significance.

**Table 1:** Differential effect of hydroxytyrosol supplementation on birth weight of male and female piglets. Mean values (±SEM) of birth-weight (kg) in female and male piglets from hydroxytyrosol-treated and control groups.

	Females	Males
**Control**	1.10 ± 0.04^A a^	1.19 ± 0.03^B c^
**Hydroxytyrosol**	1.20 ± 0.03^C b^	1.30 ± 0.03^D d^

Lowercase superscript letters denote significant differences between treatment and control groups: a≠b, 0.09 > P > 0.05; c≠d, P < 0.05. Uppercase superscript letters denote significant differences between sexes: A≠B, 0.09 > P > 0.05; C≠D, P < 0.05.

**Table 2:** Differential effect of hydroxytyrosol supplementation on birth weight of piglets from smaller or larger litters. Mean values (±SEM) of birth-weight (kg) in litters with 2–6 and 7–10 piglets in hydroxytyrosol-treated and control groups.

	Control	Hydroxytyrosol
**2–6 piglets**	1.21 ± 0.05^C^	1.38 ± 0.05^D^
**7–10 piglets**	1.13 ± 0.03^C^	1.23 ± 0.02^D^
**All litters**	1.15 ± 0.02	1.27 ± 0.02

Superscript letters denote significant differences between litter sizes: C≠D, P < 0.05.

**Fig 1:**
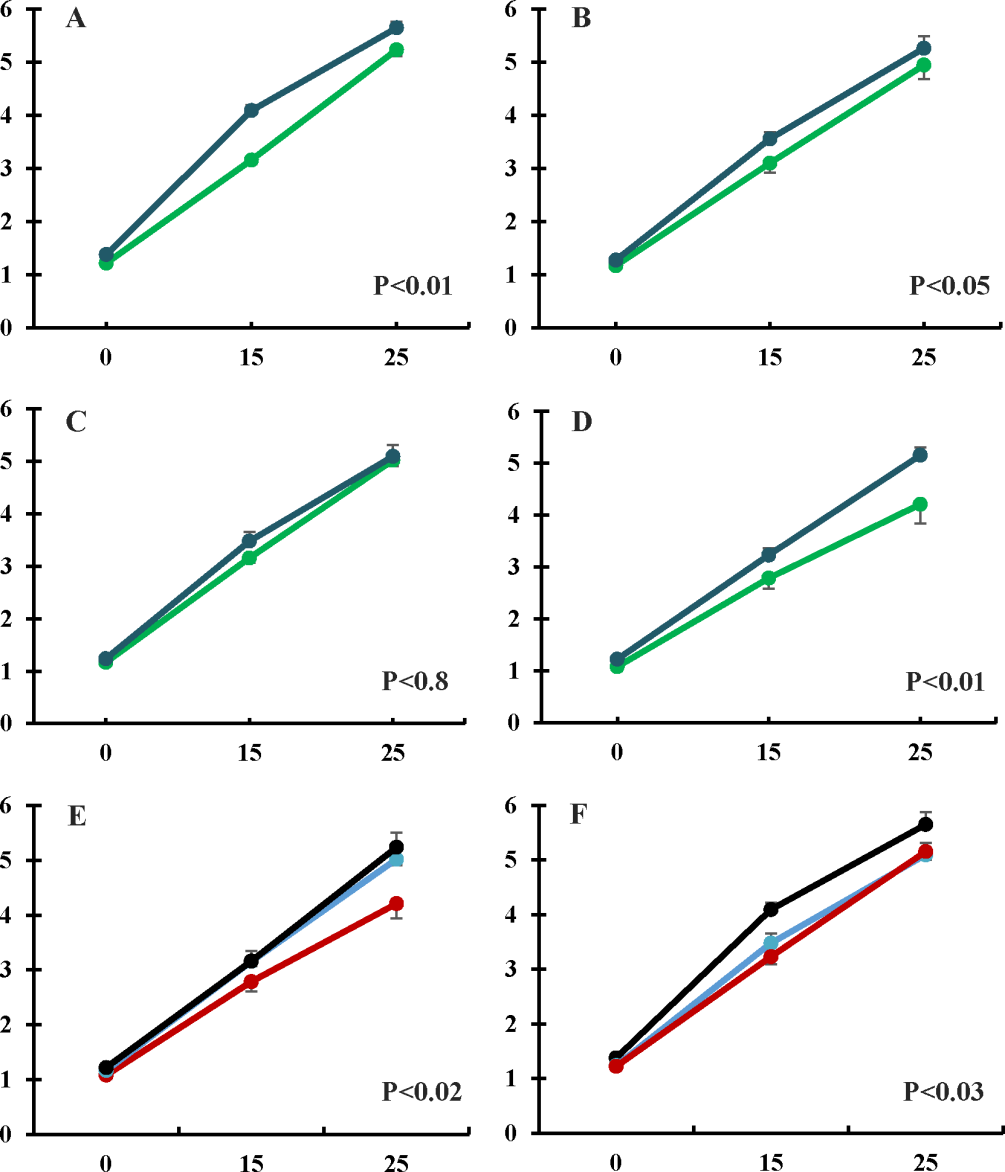
Changes in body-weight (Kg/day) of piglets throughout the lactation period. The panel A represents total differences between groups of treatment. Panels B, C and D represent between-treatments differences considering litter size (2–6, 7–8 and 9–10 piglets, respectively). Panels E and F represent within-treatment differences considering litter size in control and treated groups, respectively. Dark blue: Hydroxytyrosol group. Green: Control group. Red: 9–10 piglets/litter. Light blue: 8–7 piglets/litter. Black: 2–6 piglets/litter.

The positive effects of maternal hydroxytyrosol administration on the birth weight of piglets continued during the lactation period (Fig 1A). Piglets in the hydroxytyrosol group were significantly heavier than control piglets at 15 days (P<0.0001) and 25 days of age (P<0.05). Weight was similar between sexes within each group at each time point, but males in the group HT were significantly heavier than control males at 15 days (P<0.001) and 25 days (P<0.05) The changes in BW were significantly different between groups in piglets from the extreme litters (2–6 and 9–10 piglets; Fig 1B and 1D), but not in piglets from litters with 7–8 individuals (Fig 1C). As a result, body weight at weaning was similar for piglets in the hydroxytyrosol and control groups that were in litters with less than 9 piglets, but maternal hydroxytyrosol supplementation favored the growth of the individuals from litters of 9–10 newborns, specially between 15 and 25 days of age (P<0.01). Hence, at weaning, there was a difference of around 1Kg between these piglets in the groups HT and C and the group HT had more homogeneous body weight at weaning than the group C (Fig 1E and 1F).

**Fig 2:**
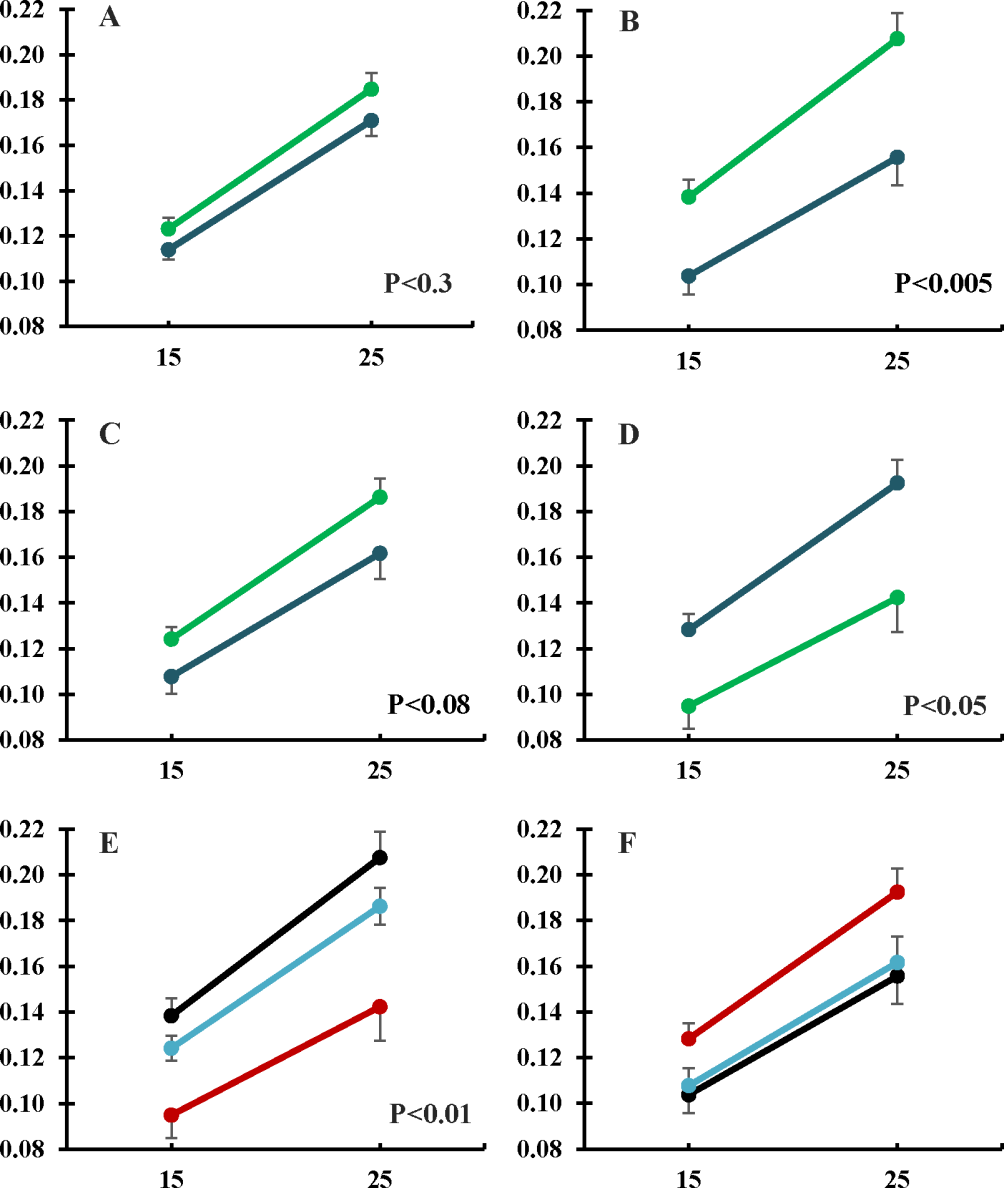
Changes in ADWG (Kg/day ± S.E.M.) of piglets throughout lactation period. The panel A represents total differences between groups of treatment. Panels B, C and D represent between-treatments differences considering litter size (2–6, 7–8 and 9–10 piglets, respectively). Panels E and F represent within-treatment differences considering litter size in control and treated groups, respectively. Dark blue: Hydroxytyrosol group. Green: Control group. Red: 9–10 piglets/litter. Light blue: 8–7 piglets/litter. Black: 2–6 piglets/litter.

The analysis of changes in the average daily weight gain (ADWG) showed concomitant changes through the lactation period. Overall, there were no significant differences in the mean value of ADWG between groups during the first 15 days. However, ADWG increased between 15 and 25 days to a greater extent in control piglets (Fig 2A), with a significant interaction between treatment and litter-size (P<0.01). These findings were related to a significant interaction among treatment, litter size and age (P<0.01). In this way, the evolution of ADWG was higher in the control group in litters with 2–6 piglets (Fig 2B), similar between treatments in litters with 7–8 piglets (Fig 2C) and higher in the group HT in litters with 9–10 piglets (Fig 2D), with greater ADWG in the group HT due to different within-litter ADWG in both groups (Fig 2E and 2F).

### Effects of hydroxytyrosol supplementation on body composition of the piglets

At weaning, hydroxytyrosol supplementation was associated with not only greater body weight but also with higher back-fat depth (4.8±0.1 *vs* 3.3±0.1 mm for piglets in the groups HT and C, respectively; P<0.01) as well as higher loin diameter (10.5±0.1 *vs* 8.9±0.1 mm for piglets in the groups HT and C, respectively; P<0.05), independently of sex and litter size.

The assessment of the relative weights of the different organs (Table 3) showed that piglets in the group C had higher relative weights of head-to-total body (P<0.0001) and brain- and liver-to-total viscerae (P<0.0001). There was again a significant interaction between treatment and litter size for the relative weights to total viscerae for kidneys, intestine and pancreas. There were only a significant difference of the relative weight of liver between groups of treatment when comparing piglets from litters with 2–6 littermates, but more ratios were higher in the group C when considering litters with 7–10 neonates, with piglets from more prolific litters in the group C having also higher relative weights of kidneys and lungs (P<0.05 and P<0.01, respectively).

**Table 3:** Ratios of organ-to-total viscera weight at weaning. Mean values (±SEM) of the relative weight (W) of organs to total viscerae-weight (TV-W) for treatment (Hydroxytyrosol, HT vs. Control group, C) and litter size (2–6 vs. 7–10 piglets/ litter).

	Total		2–6 piglets/litter		7–10 piglets/litter	
	C	HT	C	HT	C	HT
**Brain-W to TV-W**	0.06 ± 0.001	0.05 ± 0.001	0.07 ± 0.001^a^	0.06 ± 0.001^b^	0.06 ± 0.01^c^	0.04 ± 0.003^d^
**Liver-W to TV-W**	0.17 ± 0.001	0.15 ± 0.001	0.17 ± 0.01^c^	0.15 ± 0.003^d^	0.17 ± 0.01^c^	0.15 ± 0.003^d^
**Pancreas-W to TV-W**	0.01 ± 0.001	0.01 ± 0.001	0.01 ± 0.001	0.01 ± 0.001	0.01 ± 0.002	0.01 ± 0.003
**Kidneys-W to TV-W**	0.04 ± 0.001	0.03 ± 0.002	0.04 ± 0.001	0.03 ± 0.001	0.04 ± 0.002^c^	0.03 ± 0.002^d^
**Spleen-W to TV-W**	0.02 ± 0.001	0.01 ± 0.001	0.02 ± 0.001	0.02 ± 0.002	0.02 ± 0.002	0.01 ± 0.001
**Intestine-W to TV-W**	0.49 ± 0.01	0.48 ± 0.01	0.49 ± 0.02	0.47 ± 0.01	0.48 ± 0.02	0.48 ± 0.01
**Heart-W to TV-W**	0.04 ± 0.002	0.04 ± 0.002	0.04 ± 0.003	0.04 ± 0.001	0.04 ± 0.002	0.04 ± 0.002
**Lungs-W to TV-W**	0.09 ± 0.001	0.08 ± 0.001	0.10 ± 0.01^a^	0.08 ± 0.01^b^	0.09 ± 0.002^e^	0.08 ± 0.002^f^

Superscript letters indicate significant differences between groups: a≠b, 0.9 > P > 0.05; c≠d, P < 0.05; e≠f, P < 0.01.

### Effects of hydroxytyrosol supplementation on metabolic status of the piglets

Mean values for indexes of glucose and lipid metabolism at weaning were again affected by a significant in the treatment and litter size (Table 4), without sex-related effects. Among litters with 2–6 piglets, indices were similar between the hydroxytyrosol and control groups. Among litters with at least 7 piglets, plasma concentration of glucose was significantly higher in the hydroxytyrosol group (P<0.05), while the concentration of fructosamine was significantly lower (P<0.001). Again among litters with at least 7 piglets, the hydroxytyrosol group tended to show lower plasma concentrations of total cholesterol (P = 0.05), significantly lower LDL-c (P<0.05), significantly higher triglycerides (P<0.05) and similar HDL-c as the control group.

**Table 4:** Indexes of glucose and lipid metabolism in piglets at weaning. Mean plasma concentrations (±SEM) of parameters related to lipid and glucose profile (mg/dl) for treatment (Hydroxytyrosol, HT, *vs*. Control group, C) and of litter size (2–6 *vs*. 7–10 piglets/litter).

	Total		2–6 piglets/litter		7–10 piglets/litter	
	C	HT	C	HT	C	HT
**Glucose**	148.7 ± 10.5	154.2 ± 3.8	169.2 ± 29.5	152.7 ± 5.8	138.4 ± 5.4^c^	154.7 ± 4.7^d^
**Fructosamine**	312.6 ± 11.4	270.7 ± 6.9	276.2 ± 11.2	266.6 ± 15.2	330.8 ± 13.5^e^	272.2 ± 7.8^f^
**Cholesterol**	131.0 ± 7.1	113.1 ± 5.0	123.0 ± 11.6	111.3 ± 9.4	134.9 ± 9.1^a^	113.8 ± 6.0^b^
**HDL-c**	47.3 ± 2.0	49.6 ± 1.6	40.8 ± 2.7	44.9 ± 2.1	50.5 ± 2.2	51.3 ± 1.9
**LDL-c**	70.1 ± 6.4	53.8 ± 3.3	68.8 ± 8.8	54.8 ± 7.8	70.8 ± 8.9^c^	53.5 ± 3.7^d^
**Triglycerides**	58.5 ± 8.7	90.1 ± 7.0	71.2 ± 22.5	113.6 ± 14.9	52.2 ± 7.0^c^	81.7 ± 7.3^d^

Superscript letters indicate significant differences between groups: a≠b, 0.9 > P > 0.05; c≠d, P < 0.05; e≠f, P < 0.01.

## Discussion

The results of the present study support the usefulness of the maternal supplementation with hydroxytyrosol to improve prenatal development in a swine model of IUGR pregnancies. Supplementation was associated with higher mean birth weight and decreased incidence of IUGR and therefore of low-birth-weight piglets. The positive effects of hydroxytyrosol administration remained during lactation, leading to higher body-weight at weaning, especially in larger litters. It also led to deviations in body composition and metabolic indices from control piglets that suggest greater growth potential and viability.

In the current study, hydroxytyrosol supplementation during mid-to-late gestation improved pregnancy outcomes. This suggests positive effects on the feto-placental unit, since the main cause of IUGR is believed to be deficiencies in placental development and efficiency [1, 2]. Hydroxytyrosol may improve placental function through its antioxidant and immuno-modulatory effects [20, 22], since the placenta is tightly regulated by the immune-angiogenesis axis at the maternal interface [29]. Most of the data come from studies in adult humans and the information about their effects on pregnancy-related complications is still scarce [23]. Our findings suggest that hydroxytyrosol and perhaps other polyphenols may exert a range of positive influences on pre- and post-natal development, but further specific studies for confirming our hypothesis are necessary.

Polyphenols intake increases plasma antioxidant capacity in humans [23, 30] and, polyphenols supplementation has been found to improve placental oxidative stress, both *in vivo* and *in vitro* [31]. This is of paramount importance for the development of preventive and therapeutic strategies aiming to favor the adequate development of the feto-placental unit, since it is well-known that an adequate antioxidant capacity in pregnant women is related, through improvement of oxidative stress status, to alleviation of the IUGR process [32–34]. Studies linking polyphenols with health benefits have led to increased consumption of polyphenol-rich food and drinks among pregnant women, although further studies need to be undertaken [23, 35, 36] for establishing their realistic benefits, and even their potential hazards considering some evidence about adverse effects on fetal health [37] and epigenetic changes in offspring [22].

In this scenario, the results obtained in the present study support the positive effects of hydroxytyrosol supplementation on adequate pregnancy development, through alleviation of the IUGR process and increase of mean birth-weight. In swine practice, birth-weight is determinant of piglet viability and survival; low birth-weight and within-litter birth-weight heterogeneity are the most important risk factors for perinatal morbidity and mortality [38, 39]. These two problems can be particularly severe in large litters because of the competition over adequate uterine space for placental development [40, 41]. In addition, the oncoming ADWG and Feed Conversion Rate (FCR) during the fattening period are strongly correlated with these traits [27, 42], becoming a topic of major importance due to the derived economic implications in swine production. A similar situation, in the largest litters, has been found in the present study with Iberian sows (a breed with a mean prolificacy around 6.5 piglets [28]), since the newborns were heavier in non-prolific than in prolific litters (2–6 and 7–10 piglets, respectively). In spite of such difference, hydroxytyrosol was equally effective for increasing birth-weight in both prolific and non-prolific litters.

Afterwards, the benefits from hydroxytyrosol supplementation lasted during early postnatal stages, during the lactation period. At 15 days of age, mean body-weight remained higher in offspring from supplemented pregnancies than in controls; the effect was again found in piglets from both prolific and non-prolific litters, despite of fostering. Conversely, there was a significant effect of the litter size on body-weight between 15 and 25 days of age. At weaning, at 25 days of age, there were no differences in the body-weight of piglets from treated and control litters with 2–8 neonates. This result may reflect the higher ADWG of control piglets on days 15 and 25, which we are unable to explain and which deserves further study. On the other hand, hydroxytyrosol supplementation favored the ADWG and therefore, the growth of individuals from the most prolific litters (9–10 newborns). The consequence of a higher birth-weight plus a higher ADWG at weaning was a body-weight around 20% higher at weaning in these piglets than in control piglets.

We cannot elucidate, with the design of the current study, if these effects are directly related to a permanence of the effects from hydroxytyrosol supplementation, but we can hypothesize that may be more likely an indirect effect caused by higher birth-weight since higher birth-weight by itself is associated with better post-natal growth performance [9]. In any case, the benefits of maternal hydroxytyrosol supplementation for post-natal development are clear; in addition to increased body weight, supplementation was associated with greater content of subcutaneous fat and loin muscle. Prolificacy is an important trait in the pig production for its economic implications and any improvement is highly relevant. However, a higher prolificacy may be detrimental for offspring growth. Higher values of phenotypical characteristics (body-weight, back-fat depth, loin diameter) at weaning prove a good growth in the lactation period and are known to be related to a better development, and better yields, during the growing phase [43–45].

Independently of hydroxytyrosol supplementation, we observed a strong correlation between birth-weight and offspring sex, with males significantly heavier than females at birth. Nevertheless, both sexes attained similar body-weight during lactation. These data are consistent with results reported in previous work involving the same breed and dietary restriction [24]; together, these two studies support the idea that female piglets born to nutritionally restricted sows can show “catch-up growth” during lactation. We also observed evidence that piglets in the hydroxytyrosol group were better able to adapt to nutritional restriction than control neonates: We need to have in mind that catch-up growth of females during the early-postnatal period is a consequence of the prenatal restriction used in our model for increasing the incidence of IUGR processes. Hence, the findings of the current study may indicate a better adaptation to nutritional challenges of fetuses in the hydroxytyrosol group. These data are reinforced by stronger asymmetrical growth patterns in control piglets than in offspring from treated sows from favoring the development of brain, liver and other viscerae (main characteristics of nutritional IUGR; [46]).

Hydroxytyrosol supplementation also showed significant effects on glucose and lipid metabolism in the offspring, primarily in piglets from larger litters. These piglets showed lower concentrations of fructosamine (a better index than glucose itself for long periods since represents average glucose values during previous days) and lower concentrations of total and LDL cholesterol. Decreases in these two parameters were also reported in humans with cardiometabolic disorders following intake of other polyphenols [47–50]. In contrast to those human studies, hydroxytyrosol in our study was associated with higher, rather than lower, plasma concentrations of triglycerides. These results make necessary the development of further studies but we may hypothesize that the increase of triglycerides found in the HT piglets of our study may be related to their higher back-fat depth (i.e.: a higher fat deposition).

In conclusion, supplementation of maternal diet with hydroxytyrosol during pregnancy improves pre- and early post-natal developmental patterns and metabolic traits of the offspring. The major benefits are increased birth-weight, and lower incidence of IUGR and therefore of low-birth-weight piglets. In addition, in piglets from the largest litters, supplementation is associated with higher ADWG and body-weight at weaning.
